# Effectiveness of the guided self-change intervention for reducing alcohol use in adolescents with and without history of cannabis use

**DOI:** 10.3389/fpsyg.2025.1552824

**Published:** 2025-08-19

**Authors:** José Luis Carballo, Miguel Ángel Martínez-León, Ainhoa Coloma-Carmona, Virtudes Pérez-Jover, Álvaro García del Castillo-López, Clara Sancho-Domingo, Antonia Pelegrín-Muñoz, Carlos van-der Hofstadt-Román

**Affiliations:** ^1^Department of Health Psychology and Center for Applied Psychology, Miguel Hernández University, Elche, Spain; ^2^Institute for Health and Biomedical Research (ISABIAL), Alicante, Spain; ^3^Health Psychology Unit, Dr. Balmis General University Hospital, Alicante, Spain

**Keywords:** brief intervention, guided self-change program, substance use, adolescents, school setting, alcohol, substance use profile

## Abstract

**Introduction:**

The use of alcohol is a prevalent phenomenon among adolescents. Several brief intervention strategies have been developed to prevent the progression of alcohol use to high-risk levels. The consumer profile, including whether they have consumed one or more substances, may be a key variable in analyzing the effectiveness of interventions.

**Methods:**

This study consists in a secondary analysis of data from a randomized controlled trial (PREVENALC) to examine the effectiveness of a brief intervention based on the Guided Self-Change Program (GSC) and its interaction with substance use profile. Participants were the 629 students in the experimental arm of PREVENALC who received the GSC and completed the pretest, posttest, and 6-moth follow-up measures. The sample was categorized into two groups according to substance use profile: Group of Alcohol Users (GA) (*n* = 438, 76.8%) and Group of Alcohol and Cannabis Users (GAC) (*n* = 144, 23.2%). Alcohol consumption (Standard Drinking Units (SDUs), binge episodes and days of abstinence) and problematic drinking were considered as primary variables. Readiness to change and self-efficacy were considered as secondary variables.

**Results:**

Overall, GAC students demonstrated higher rates of alcohol consumption and lower levels of readiness to change at baseline. Both groups reduced the total amount of alcohol, binge drinking episodes and problematic drinking and increased the percentage days of abstinence in the last month. Nevertheless, GAC reduced the amount of alcohol in the last month more significantly than GA. However, the GAC did not maintain improvements in terms of the percentage of days of abstinence in the medium term.

**Discussion:**

Future research should consider substance use profiles when evaluating the efficacy of interventions. Furthermore, large sample designs, control groups and longer follow-ups are required.

## 1 Introduction

Over the past decade, the prevalence of drug use has increased by 20%, with alcohol and cannabis being the most commonly used psychoactive substances worldwide (United Nations Office on Drugs and Crime, 2024). In Spain, up to 73.6% of students aged 14 to 18 years report having consumed alcohol in the last 12 months, and 28.2% of students have engaged in binge drinking in the last month. Cannabis use is also common in this population, with 21.8% reporting past-year use and 15.6% past-month use. The average age of onset is 13.9 years for alcohol and 14.9 years for cannabis (Observatorio Español de las Drogas y las Adicciones, 2023).

The concurrent use of alcohol and cannabis is a prevalent phenomenon among the youth population (Gonçalves et al., 2023; Hai et al., 2022). Similar to trends observed in other countries (Keyes et al., 2022), in Spain, 13.7% of students reported using both alcohol and cannabis in the previous year. Furthermore, up to 35.8% of students who reported alcohol use in the last month also used cannabis (Observatorio Español de las Drogas y las Adicciones, 2023). This pattern is particularly pronounced among students who engage in more hazardous forms of alcohol consumption, such as binge drinking (Vijapur et al., 2021). Data from national surveys indicate that past-year cannabis use is five times more prevalent among those who participate in “botellón” activities (Observatorio Español de las Drogas y las Adicciones, 2023), which is a common youth practice in Spain characterized by drinking in public settings and is often associated with heavy episodic drinking (García-Couceiro et al., 2020; García-Couceiro et al., 2023).

Combined use of alcohol and cannabis represents a higher risk profile than the use of a single substance, increasing the consumption rates of both substances and the risk of developing a substance use disorder (Karoly et al., 2020; Linden-Carmichael and Wardell, 2021; Rodríguez-Ruiz et al., 2021; Subbaraman and Kerr, 2015). In addition, co-use worsens the negative consequences associated with alcohol and cannabis use such as dizziness, blackouts, poor academic performance, and increases involvement in criminal activity (Carbonneau et al., 2023; Davis et al., 2021; Hammond et al., 2020; Rodríguez-Cano et al., 2023; Stevens et al., 2022).

Consequently, selective prevention strategies are needed to recognize these risk profiles and act early to prevent the progression to higher-risk consumption (López Pelayo and Cortés-Tomás, 2021). Accordingly, there has been a growing scientific interest in studying the effectiveness of Brief Intervention (BI) models that offer numerous advantages in terms of cost-effectiveness and reach (McCambridge, 2021).

There are many definitions and types of BI. Generally, BIs are brief (1–5 sessions) and implement motivational strategies, coping skills training, and relapse prevention (Carballo and Coloma-Carmona, 2025; Coloma-Carmona and Carballo, 2022; Evans et al., 2011; Mattoo et al., 2018). In both young and adult populations, BIs have been demonstrated to be effective in reducing alcohol consumption (Mcqueen et al., 2015; Tanner-Smith et al., 2022), tobacco use (Hartmann-Boyce et al., 2021) and cannabis consumption (Halladay et al., 2019; Li et al., 2019). Evidence supports the effectiveness of BIs in diverse settings such as primary care and emergency departments (Barbosa et al., 2020; Kaner et al., 2018; Tanner-Smith et al., 2022).

The evidence on the efficacy of BIs in reducing substance use in school settings remains limited and inconsistent (Carballo and Coloma-Carmona, 2025). Nevertheless, some studies suggest that BIs can be beneficial for adolescents. The meta-analysis conducted by Tanner-Smith and Lipsey (2015) indicated that BIs were associated with a significant reduction in alcohol consumption (*g* = 0.27) and alcohol-related problems (*g* = 0.19) among adolescents. Similarly, Carney et al. (2016) concluded in their meta-analysis that BIs may be an effective approach for reducing substance use in school settings when compared to control groups that were only evaluated. Their results demonstrate that BIs markedly reduced the frequency of alcohol (SMD = –0.91; 95% CI = –1.21 to −0.61) and cannabis (SMD = –0.83; 95% CI = –1.14 to −0.53) use in the short term. Likewise, Hogue et al. (2018) argue that BIs may prove an efficacious intervention in adolescents. Nevertheless, the majority of studies have not considered the influence of different substance use profiles in their analyses (Carney et al., 2016; Hogue et al., 2018; Tanner-Smith and Lipsey, 2015), which may critically influence intervention outcomes (Abedi et al., 2019). This is particularly relevant given that the co-use of cannabis and alcohol is common and can contribute to the development of more severe substance use profiles (Linden-Carmichael and Wardell, 2021).

Among BIs, those incorporating a strong motivational interviewing approach appear to be the most effective in young populations (Gex et al., 2024; Kohler and Hofmann, 2015; Merz et al., 2015). The Guided Self-Change (GSC) program is an example of this type of BI. The GSC employs motivational interviewing as a fundamental component, integrating it with cognitive-behavioral techniques and relapse prevention strategies. The GSC is comprised of four sessions and can be delivered in both individual and group formats (Sobell and Sobell, 2011). The program has been adapted for the Spanish speaking population and has demonstrated effectiveness in reducing substance use (Carballo et al., 2020; Martínez Martínez et al., 2010). It has also demonstrated efficacy in adolescents. Wagner et al. (2014) found that the GSC was effective in reducing the number of days of alcohol use (*d* = 0.45) and other drug use (*d* = 0.22) among students. Furthermore, a recent study found that the GSC was also effective in increasing the self-efficacy levels of college students who used alcohol and cannabis (Langwerden et al., 2023).

Despite these promising findings, the existing literature emphasizes the need for additional studies to assess the effectiveness of BI models, such as the GSC, in school-based settings for the prevention of substance use (Halladay et al., 2019; Vázquez et al., 2018). Moreover, there is a need for research that explores whether patterns of substance use that are common among adolescents, such as alcohol and cannabis use, may influence intervention outcomes. Addressing this gap could strengthen the evidence base on the effectiveness of BI approaches for adolescents with diverse substance use profiles. To this end, the present study examined data from participants in the intervention arm of the PREVENALC randomized trial, which aimed to evaluate the efficacy of the GSC program in reducing alcohol use among adolescents aged 16–18 years. Specifically, it explored whether the GSC effectiveness differed between those who reported alcohol use only and those with a history of cannabis use.

## 2 Method

### 2.1 Study design and procedure

This study is part of the PREVENALC project, which is registered in ClinicalTrials.gov (Protocol Record ID: NCT05281172), and was conducted in accordance with CONSORT guidelines for Randomized Clinical Trials (Cobos-Carbó, 2005). The project received ethical approval from the Ethics Committee for Drug Research of the Alicante Department of Health - General Hospital (CEIm; PI2019/112).

PREVENALC was a randomized controlled trial designed to evaluate the efficacy of the GSC Program in reducing alcohol use among adolescents aged 16 to 18 years. The study was conducted between 2022 and 2024 in secondary schools in the province of Alicante (Spain). All procedures were carried out during regular school hours.

Prior to initiate contact with the educational institutions, authorization was obtained from the Department of Education of the Valencian Community. A report was drafted and submitted via mail to the relevant center personnel, requesting their participation in the study. A total of 30 secondary high schools were randomly selected in the province of Alicante, of which 16 agreed to participate in the study. These schools were randomly allocated to experimental or control conditions. Informative meetings were held with each participating school to coordinate implementation, and commencement dates were scheduled for both the assessment and intervention phases.

A double-blind design was employed, whereby neither the participants nor the health psychologists who delivered the intervention were aware of group assignment. All participants were informed that they would be participating in a program related to substance use. The assessment and intervention phases were conducted over a period of 5 weeks at each secondary school: 1 week for baseline assessment and 4 weeks of intervention. Each session lasted approximately 60 min. Assessments were completed using an online platform developed specifically for the PREVENALC project, allowing adolescents to complete the questionnaires via their mobile phones. When the use mobile devices of was not feasible, paper-based versions of the questionnaires were provided. During the intervention phase, students in the experimental group received the GSC program, while the control group was provided with information regarding healthy habits.

### 2.2 Participants

Eligibility criteria for inclusion in the study were as follows: (1) providing informed consent, with one form signed by the adolescent and another by their legal guardian (or by the adolescents themselves when they reach the age of majority); (2) aged between 16 and 18 years old and beginning 11th grade or an equivalent vocational training cycle; and (3) reported alcohol use at least once in the past year. Exclusion criteria were: (1) undergoing psychological or psychiatric treatment currently; (2) diagnosis of a severe mental disorder; and (3) presence of other substance dependence disorders.

*A priori* sample size calculations were performed to ensure sufficient statistical power. Given the total student population of approximately 25,000 in the study region and an estimated prevalence of regular alcohol use of 56.6% among Spanish adolescents (Observatorio Español de las Drogas y las Adicciones, 2023), along with sample sizes reported in previous school-based studies (Carney et al., 2016; Halladay et al., 2019), the minimum required sample size was estimated at 372 participants per group (95% confidence level, 5% margin of error).

**Figure 1:**
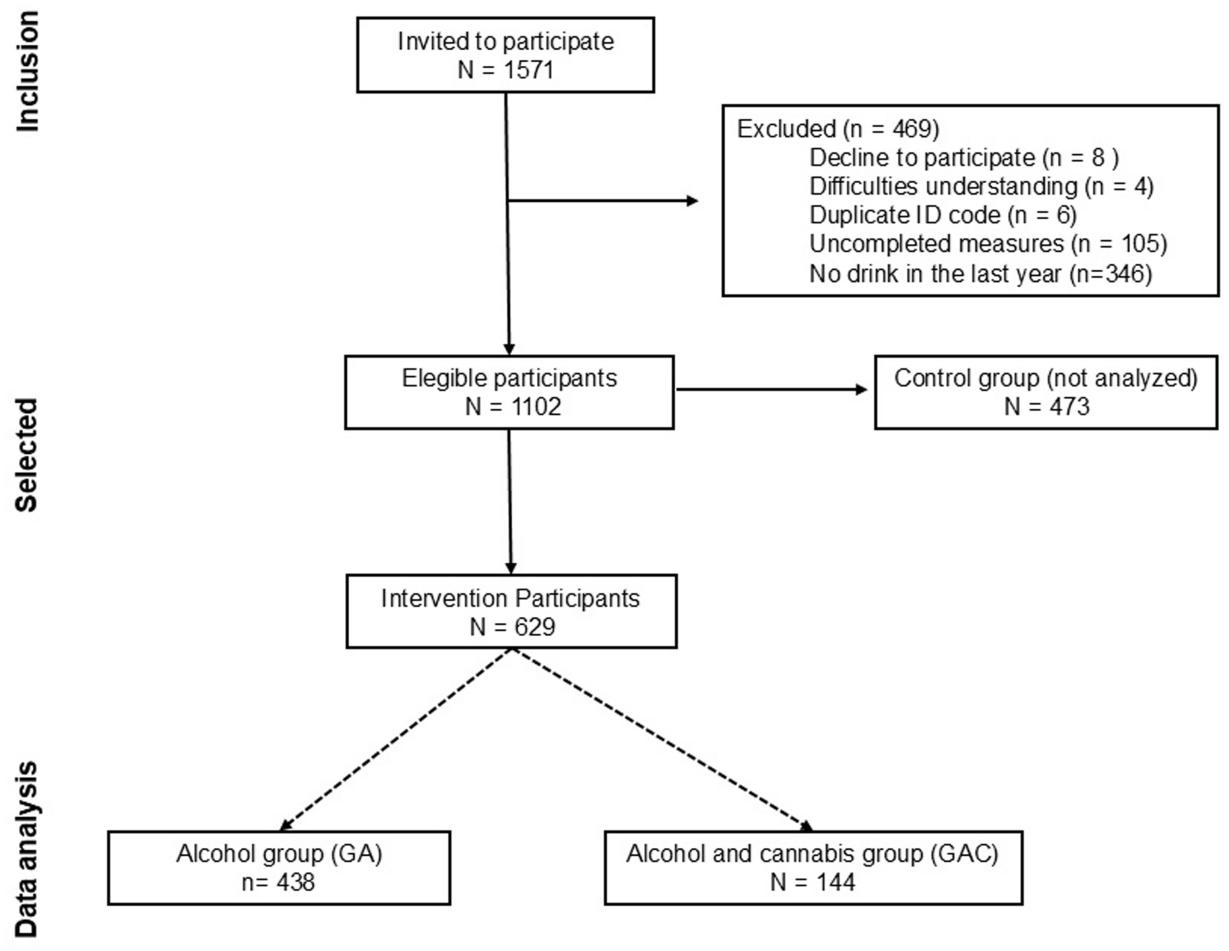
Flow diagram for study participants.

A total of 1,571 students from the 16 participating schools were enrolled in the PREVENALC trial (Figure 1). Of these, 469 students were excluded from analyses for not meeting eligibility criteria. The final sample included *N* = 1,102 adolescents, distributed between the experimental group (*n* = 629), which received the GSC intervention, and the control group (*n* = 473). The present study focuses on the subsample from the experimental arm (*n* = 629) who completed all three assessments: pretest, posttest, and 6-month follow-up.

To explore whether intervention outcomes varied based on substance use profiles, participants were categorized into two subgroups based on their self-reported substance use in the 12 months prior to the pretest assessment: (1) adolescents who reported alcohol use only (GA; 76.8%, *n* = 438), and (2) adolescents who reported both alcohol and cannabis use (GAC; 23.2%, *n* = 144).

Of the participants, 56.4% (*n* = 355) were female, with a mean age of 16.34 ± 0.79 years. The two groups were found to be homogeneously distributed regarding the variables of sex (χ^2^ = 0.317, *p* > 0.05, *φ* = 0.02) and age (*F* = –1.092, *p* > 0.05, *d* = 0.11).

### 2.3 Intervention (the GSC)

Participants received a BI based on the GSC program, applied in group format (Sobell and Sobell, 2011). The intervention was conducted in four weekly sessions, each lasting 60 min, at each of the participating study centers.

(1) Session 1: Group norms are established. Problematic alcohol use psychoeducation, explanation of self-reporting tasks, and guidance on identifying high-risk situations are provided. Decisional balance exercise is carried out and participants set their goals to promote a change in consumption patterns; (2) Session 2: The results of the questionnaires and self-monitoring records are reviewed and discussed. Feedback is provided regarding the severity profile and the high-risk situations experienced by the participants. Psychoeducation is conducted about students’ consumption in the last month and guiding questions are posed to identify risk situations. Another decisional balance exercise and relapse prevention strategies psychoeducation is carried out; (3) Session 3: The results of the self-reporting records are reviewed again. An exercise on risk situations and coping skills is conducted and action plans are developed for each risk situation. The “Motivational Ruler Chart” exercise is conducted providing feedback on the results so far. For the next session, participants are asked to complete an exercise on risk situations and self-efficacy. They must indicate how they respond to different situations and assess their level of self-efficacy; (4) Session 4: Final review and feedback of all materials and tasks completed during the week are conducted and the action plans are subjected to a review. A comparison of the students’ results obtained after a 5-week period is made. The “Ready to Change” chart is employed for providing feedback and encourage reflection. The post-test evaluation is administered, and the session concludes by reinforcing positive changes and expressing gratitude for participation.

### 2.4 Measures

#### 2.4.1 Sociodemographic data

Sociodemographic data were collected, including age, sex, and grade.

#### 2.4.2 Primary variables

Alcohol consumption (Standard Drink Units, binge episodes and days of abstinence).

The Spanish translation and cultural adaptation of the TimeLine Follow-Back (TLFB; Sobell and Sobell, 1992) were used to assess alcohol consumption. The tool comprises a calendar that records the number of Standard Drink Units (SDUs) consumed in the last month. In Spain, a SDU is defined as 10 grams of alcohol (Rodríguez-Martos Dauer et al., 1999). Each box on the calendar corresponds to a day in the previous month and should be completed by noting the number of SDUs consumed on that day. The data were used to calculate the total number of SDUs and the percentage of days of abstinence from alcohol use in the last month. The TLFB was also employed to collect binge drinking episodes in the last month. Binge drinking episodes are defined as the consumption of 5 or more SDUs for men and 4 or more SDUs for women within 2 h (National Institute on Alcohol Abuse and Alcoholism, 2024). The total number of binge episodes in the past month was calculated. Although there are no formal validation studies in Spanish-speaking populations, international research has demonstrated that the TLFB exhibits good concurrent validity. The TLFB correlates significantly with standardized alcohol use measures as the AUDIT (*r* = 0.32 to 0.41; Sobell and Sobell, 2003). The scale has demonstrated reliability and validity in adolescent populations, both in clinical settings (Dennis et al., 2004) and in non-clinical samples of adolescents using online administration, with high concordance across formats (*r* = 0.86–0.94; Hareskov Jensen et al., 2023).

##### 2.4.2.1 Problematic drinking

The Spanish version of the Alcohol Use Disorders Identification Test (AUDIT; Rubio Valladolid et al., 1998; Saunders et al., 1993) was employed to assess problematic drinking. The scale comprises 10 items, each of which is scored from 0 to 4, thus allowing for a total possible score of 40. A higher score is indicative of a greater degree of problematic alcohol use. The AUDIT was administered solely at the pre-intervention stage and at the 6-month follow-up, given that a longer period is required for reassessment. The tool has demonstrated satisfactory reliability indices (*α* > 0.75) in adolescent populations (Cortés-Tomás et al., 2016; Hallit et al., 2020).

##### 2.4.2.2 Cannabis use

The use of cannabis was confirmed through the Drug History Questionnaire (DHQ; Sobell et al., 1995). The adapted and Spanish translation version was used (Carballo et al., 2014). Specifically, the item “Have you ever used cannabis in the last 12 months?” was used to identify adolescents with past-year cannabis use among those who reported alcohol consumption. The DHQ has demonstrated adequate psychometric properties, with test–retest reliability ranging from moderate to high (intraclass an Pearson correlation coefficients between *r* = 0.53 to *r* = 0.93; Sobell et al., 1995).

#### 2.4.3 Secondary variables

##### 2.4.3.1 Readiness to change

The readiness to change was evaluated through the one-item version of the Readiness to Change Ruler (Miller, 1999). Scores range from 1 “I am not at all ready to change” to 10 “I am completely ready to change.” The scale has demonstrated strong reliability and both concurrent and predictive validity when used with adolescent clinical populations (Maisto et al., 2011).

##### 2.4.3.2 Self-efficacy

The Brief Situational Confidence Questionnaire (BSCQ; Breslin et al., 2000) was employed to assess alcohol-related self-efficacy. Consist of a scale ranging from 0 (“not all confident”) to 100% (“totally confident”) to evaluate the extent to which participants feel confident, at the present time, of refraining from alcohol consumption in situations deemed risky. The BSCQ includes 8 items with high-risk situations such as social pressure, negative physical states, or positive emotional states. The tool has demonstrated satisfactory psychometric properties, with a Cronbach’s α value exceeding 0.84 in adolescent populations (Delaney et al., 2020).

### 2.5 Data analysis

All statistical analysis were conducted using IBM SPSS Statistics program, version 26.0. Descriptive statistics (percentages, frequencies, and means) were calculated in order to examine the characteristics of the sample. To examine differences in pretest measures between GA and GAC groups, the chi-square test was employed for non-continuous variables and t-test for continuous variables. Effect sizes were calculated with Phi *φ* (0.1 = small; 0.30 = moderate; 0.5 = large) for non-continuous variables and Cohen’s d (0.20–0.49 = small; 0.50–0.79 = moderate; >0.80 = large) for continuous variables (Cohen, 1988).

A mixed repeated-measures analysis of variance (ANOVA) was conducted to assess differential responses to the intervention over time between the two consumption profiles. The model included two factors: time (pretest, posttest, and 6-month follow-up) as a within-subject factor, and consumption profile (GA vs. GAC), as a between-subjects factor. The main effects were analyzed to assess overall changes over time and differences between GA and GAC participants, regardless of time point or group. Subsequently, the interaction effect between both factors was analyzed to determine whether the two profiles exhibited different patterns of change following the intervention. Post-hoc comparisons were performed using the Bonferroni correction to explore the significant differences between specific time points.

The Mauchly test was employed to test the hypothesis of sphericity, and the Greenhouse–Geisser correction was utilized if it was violated. To calculate the effect size, n^2^ (0.01 = small; 0.06 = moderate; 0.14 = large) was employed (Cohen, 1988). A confidence level of 95% was utilized throughout the data analysis phase.

## 3 Results

### 3.1 Demographics and group characteristics

#### 3.1.1 Primary outcomes

##### 3.1.1.1 Alcohol consumption

Regarding the total consumption of SDUs over the past month, students in the GAC group consistently reported higher levels of consumption across all evaluated time points compared to those in the GA group (Group: *F* = 48.33, *p* < 0.001, η^2^ = 0.12). The within-subject analysis indicated a statistically significant reduction in the number of SDUs consumed from the pre-intervention assessment (*M* = 11.7 ± 17.06) to the intervention period (*M* = 4.6 ± 9.08) and the 6-month follow-up period (*M* = 13.6 ± 19.63) in both groups (Time: *F* = 45.4, *p* < 0.001, η^2^ = 0.12). However, the pattern of reduction differed between groups (Time*Group: *F* = 5.83, *p* = 0.005, η^2^ = 0.02), with a more pronounced decrease observed in the GAC group (Pretest: *M* = 21.6 ± 23.14, Intervention: *M* = 9.4 ± 14.10) compared to the GA group (Pretest *M* = 9.0 ± 13.88, Intervention: M = 3.2 ± 6.57) (Table 1). At follow-up, both groups increased the number of SDUs (GA: *M* = 5.3 ± 10.21; GAC: *M* = 13.6 ± 19.63), but were still lower than at pretest (*p* < 0.01).

**Table 1:** ANOVA results for alcohol consumption.

Variables	GA M ± SD (*n* = 438)			GAC M ± SD (*n* = 144)					
	Pretest Mean ± SD	Posttest Mean ± SD	6 months Mean ± SD	Pretest Mean ± SD	Posttest Mean ± SD	6 months Mean ± SD	Time	Group	Time*Group
Total SDUs last month	9.0 ± 13.87^a^	3.2 ± 6.57^b^	5.3 ± 10.21^c^	21.6 ± 23.14^a^	9.4 ± 14.10^a^	13.6 ± 19.63^c^	*F* = 45.45**, η^2^ = 0.12	*F* = 48.33**, η^2^ = 0.12	*F* = 5.83**, η^2^ = 0.02
Percentage of days of abstinence, last month	94.7 ± 7.24^a^	98.0% ± 4.07^b^	96.5 ± 5.88^c^	90.3 ± 8.61^a^	96.0% ± 4.62^b^	92.5 ± 10.53^a^	*F* = 49.76**, η^2^ = 0.13	*F* = 27.26**, η^2^ = 0.07	*F* = 4.20*, η^2^ = 0.01
Binge drinking episodes, last month	0.7 ± 1.31^a^	0.2 ± 0.5^b^	0.5 ± 1.01^a^	1.4 ± 1.62^a^	0.7 ± 1.06^b^	1.1 ± 1.75^a^	*F* = 21.73**, η^2^ = 0.06	*F* = 30.47**, η^2^ = 0.08	*F* = 0.64, η^2^ = <0.01
Problematic drinking (AUDIT scores)†	4.9 ± 3.89^a^	–	4.5 ± 3.17^b^	8.7 ± 4.9^a^	–	7.7 ± 4.28^b^	*F* = 7.72**, η^2^ = 0.02	*F* = 72.39**, η^2^ = 0.17	*F* = 1.43, η^2^ = <0.01

M: Mean; SD: Standard Deviation; *p < 0.05, **p < 0.01. GA: Alcohol users group; GAC: Alcohol and cannabis users group.^a, b, c^Indicate differences in *post-hoc* comparisons. Letter changes indicate statistically significant differences between values. †Participants who did not complete the AUDIT were excluded (*n* = 239).

##### 3.1.1.2 Percentage of days of abstinence

Participants in the GAC group showed significantly lower percentages of days of abstinence from alcohol use compared to students in GA group on all assessments (Group: *F* = 27.26, *p* < 0.001, η^2^ = 0.07) (Table 1). In addition, a significant main effect of Time was observed (*F* = 49.76, *p* < 0.001, η^2^ = 0.13), indicating that overall, participants in both groups showed significant changes in abstinence across assessments. However, a significant effect of the Time*Group interaction was observed (*F* = 4.2, *p* = 0.018, η^2^ = 0.01), reflecting a different progression between the two groups evaluated. In the GA group, the abstinence percentage increased significantly from the pretest evaluation (*M* = 94.7% ± 7.24%) to the intervention (*M* = 98% ± 4.07%) and remained elevated at follow-up (*M* = 96.5% ± 5.88%). In contrast, GAC group increased from the pretest (*M* = 90.3% ± 8.61%) to the intervention (*M* = 96% ± 4.62%), returning to levels similar to pretest (*M* = 92.5% ± 10.53%).

##### 3.1.1.3 Binge drinking episodes

Although the amount of binge drinking episodes in the past month was higher in GAC group compared to GA group (Group: *F* = 30.5, *p* < 0.001, η^2^ = 0.08), both groups statistically significantly reduced the number of binge drinking episodes at intervention, but not at follow-up (Time: *F* = 21.73, *p* < 0.001, η^2^ = 0.06) (Table 1). Both groups statistically significantly decreased the total number of binge drinking episodes from pretest (GA: *M* = 0.7 ± 1.31; GAC: *M* = 1.3 ± 1.62) to intervention (GA: M = 0.2 ± 0.5; GAC: M = 0.7 ± 1.06), but returned to levels close to those at baseline assessment at follow-up (GA: *M* = 0.5 ± 1.01; GAC: *M* = 1.1 ± 1.75). There was no statistically significant interaction effect between time and participant group (*F* = 0.64, *p* = 0.506, η^2^ = 0.01).

##### 3.1.1.4 Problematic drinking (AUDIT total scores)

The GAC group showed greater rates of problematic drinking at the two times evaluated compared to the GA group (Group: *F* = 72.4, *p* < 0.001, η^2^ = 0.17) (Table 1). The within-subjects analyses revealed a statistically significant effect of the Time factor (*F* = 7.71, *p* = 0.006, η^2^ = 0.02), indicating that both groups reduced the rate of problematic drinking from pretest (GA: *M* = 4.9 ± 3.89; GAC: *M* = 4.5 ± 3.17) to 6-month follow-up (GA: *M* = 8.7 ± 4.9: GAC: *M* = 7.7 ± 4.28). No statistically significant interaction effect was found between time and substance use profile group (*F* = 1.4, *p* = 0.232, η2 = 0.01).

### 3.2 Secondary outcomes

#### 3.2.1 Readiness to change

GAC group showed significantly lower levels of readiness to change compared to the students in GA group on all assessments (Group: *F* = 7.00, *p* = 0.009, η^2^ = 0.02). A significant main effect of Time (*F* = 12.63, *p* < 0.001, η^2^ = 0.04) was observed. Participants in GA group increased readiness to change between the pretest (*M* = 7.6 ± 3.26) to intervention (*M* = 8.5 ± 2.44) but returned to levels similar to the pretest at follow-up (*M* = 7.15 ± 3.43). GAC group showed an increase readiness to change from pretest (*M* = 6.8 ± 3.15) to intervention (*M* = 7.8 ± 2.68) but it was not statistically significant. At follow-up, the levels again decreased statistically significantly (*M* = 6.3 ± 3.23). There was no statistically significant interaction effect Time*Group (*F* = 0.08, *p* = 0.911, η^2^ = 0.01) (Table 2).

**Table 2:** ANOVA Results for readiness to change and self-efficacy.

Variables	GA M ± SD (*n* = 438)			GAC M ± SD (*n* = 144)					
	Pretest Mean ± SD	Posttest Mean ± SD	6 months Mean ± SD	Pretest Mean ± SD	Posttest Mean ± SD	6 months Mean ± SD	Time	Group	Time*Group
Readiness to change	7.6 ± 3.26^a^	8.5 ± 2.44^b^	7.1 ± 3.43^a^	6.8 ± 3.15 ^ab^	7.8 ± 2.68^a^	6.3 ± 3.23^b^	*F* = 12.63**, η^2^ = 0.04	*F* = 7.00*, η^2^ = 0.02	*F* = 0.08, η^2^ = 0.01
Self-efficacy BSCQ	80.5 ± 15.45^a^	86.3 ± 14.4^b^	83.3 ± 15.96^ab^	73.5 ± 19.3 ^a^	85.5 ± 14.4^b^	82.1 ± 18.25^b^	*F* = 15.77**, η^2^ = 0.09	*F* = 1.37, η^2^ = 0.01	*F* = 2.35, η^2^ = 0.01

M: Mean; SD: Standard Deviation; *p < 0.05, **p < 0.01. GA: Alcohol users group; GAC: Alcohol and cannabis users group. ^a, b, c^Indicate differences in post-hoc comparisons. Letter changes indicate statistically significant differences between values.

#### 3.2.2 Self-efficacy

In relation to self-efficacy, scores were similar between groups at all time points (Group: *F* = 1.37, *p* = 0.243, η^2^ = 0.01). Both groups experienced a statistically significant increase from pretest (GA: *M* = 80.5 ± 15.45; GAC: M = 73.5 ± 19.3) to intervention (GA: *M* = 86.3 ± 14.4; GAC: *M* = 85.5 ± 14.48) (Time: *F* = 15.77, *p* < 0.001, η^2^ = 0.09). However, although self-efficacy levels decreased again at follow-up, the GAC group maintained significantly higher levels than in the pretest (*M* = 82.1 ± 18.25). In contrast, the GA group returned to levels that were comparable to their pretest levels (*M* = 83.3 ± 15.96). No statistically significant interaction effect was found between time and group (*F* = 2.35, *p* = 0.100, η^2^ = 0.01) (Table 2).

## 4 Discussion

This study aimed to address the limited evidence on the effectiveness of brief interventions in school settings, particularly among adolescents with different substance use profiles. Specifically, a secondary analysis was conducted on participants assigned to the intervention arm of the PREVENALC randomized controlled trial to evaluate whether a history of cannabis use moderated the effectiveness of the GSC program in reducing alcohol use among Spanish adolescents aged 16–18.

Our findings indicate that implementing the GSC program in educational settings is an effective strategy for reducing alcohol consumption among adolescents, including those with more complex substance use profiles. Both groups exhibited significant short-term improvements after the intervention, including reductions in the total amount of SDUs, fewer binge drinking episodes, and increases in abstinence days. These behavioral changes were accompanied by increases in self-efficacy and readiness to change, key mechanisms of behavior change (Bandura, 1977; Kelly and Greene, 2014) that have been consistently linked to lower alcohol use (DiBello et al., 2019; Gázquez Linares et al., 2023; Walsh et al., 2024).

These findings are consistent with the results of earlier research, which highlighted the mediating role of these variables in the effectiveness of motivational interventions such as the GSC, across both adult (Sancho-Domingo et al., 2024) and adolescent populations (Magill et al., 2017). Additionally, they reinforce prior evidence on the program’s efficacy in reducing alcohol and other drug use among adolescents and young adults (Gil et al., 2004; Langwerden et al., 2023; Wagner et al., 2014), and further extend this evidence by demonstrating its effectiveness when implemented within school settings.

Importantly, our findings also show that the program benefits adolescents regardless of whether they have initiated cannabis use. At pretest, the GA and GAC groups differed significantly in their patterns of alcohol use and in key mechanisms of behavior change. Adolescents who reported past-year cannabis use exhibited a more severe profile, with higher levels of alcohol consumption, both in total SDUs and binge drink episodes, fewer abstinent days in the past month, and lower scores in alcohol-related self-efficacy and readiness to change. This finding aligns with prior research indicating that adolescents who engage in high-risk drinking and cannabis use often report reduced motivation to change and lower confidence in their ability to resist drinking in high-risk situations (Sancho-Domingo et al., 2025). It also supports evidence indicating that the early initiation of multiple substance use can contribute to the development of more severe patterns of substance use (Davis et al., 2021; Halladay et al., 2020; Stevens et al., 2022; Subbaraman and Kerr, 2015).

Despite these initial group differences, adolescents in the GAC group demonstrated short-term improvements that were comparable to, or even exceeded those observed in the GA group. Specifically, the GAC group showed a steeper short-term reduction in alcohol use, particularly with regard to reported SDUs and percentage days of abstinent days. These behavioral changes were accompanied by significant improvements in the mechanisms of change associated with motivational interventions. Despite initial lower levels of self-efficacy and readiness to change among the GAC group, posttest scores were comparable to those of the GA group, suggesting that the GSC program effectively enhanced these key mechanisms even among participants with more severe substance use profiles.

Regarding self-efficacy, it is noteworthy that only the GAC group exhibited statistically significant within-group improvements in self-efficacy from the pretest to the 6-month follow-up. However, this outcome is likely attributable to their initially lower baseline scores rather than a differential effect of the program. Altogether, our findings suggest that the GSC program can produce sustained gains in key motivational variables across adolescents with differing levels of severity, supporting its potential utility for adolescents with more severe substance use patterns.

Nevertheless, despite the notable short-term improvements, not all outcomes remained significantly improved at follow-up. While both groups demonstrated a significant decrease in AUDIT scores at the 6-month follow-up compared to the pretest, which indicates a reduction in problematic alcohol use, the number of abstinent days only remained significantly increased in the GA group. Furthermore, while the decline in SDUs was maintained across both groups, adolescents in the GAC group continued to report elevated levels of overall alcohol consumption. This pattern is consistent with previous research indicating that polysubstance use contributes to increased clinical complexity and is associated with poorer treatment outcomes among adolescents (Crummy et al., 2020; Karoly et al., 2020). It also aligns with studies indicating that brief motivational interventions tend to be more effective in reducing overall consumption rather than in promoting sustained abstinence (Winters et al., 2014).

Moreover, both groups displayed a reversion to their pre-intervention levels of binge drinking at the 6-month follow-up. In this regard, previous studies have indicated that brief interventions tend to be more effective for behaviors that adolescents perceive as riskier (Winters et al., 2014). In Spain, 30% of secondary school students report engaging in binge drinking, a pattern of alcohol use that is especially common in “botellón,” a form of social drinking reported by 50% of Spanish adolescents aged 14 to 18 (Observatorio Español de las Drogas y las Adicciones, 2023). In light of the normalization of this practice among youth, these findings suggest that while the GSC program may serve as a valuable strategy to initiate change, additional or booster sessions might be necessary to consolidate and sustain reductions, particularly in high-risk or more socially normalized patterns of alcohol use (Winters et al., 2011).

To date, the majority of the studies that examine the effectiveness of BIs do not consider the substance use profile as a variable that may influence the results obtained (Carney et al., 2016; Hogue et al., 2018; Tanner-Smith and Lipsey, 2015). However, the analyses of this study indicate that the substance use profile may be a factor influencing the effectiveness of the intervention. This would help to explain the inconsistency of results regarding the efficacy of BIs in reducing substance use (Carney et al., 2016; Halladay et al., 2019).

This study presents several methodological limitations that should be considered when interpreting the findings. First, the use of self-report measures may introduce response biases, such as social desirability, misinterpretation of items, or inattentive responding. To mitigate these risks, several procedural safeguards were implemented to enhance data quality. The assessment sessions were conducted under supervision of trained therapists, who would clarify items and ensure a standardized administration. Additionally, questionnaire order was counterbalanced across participants to minimize peer influence. Despite these precautions, the self-efficacy scale yielded a considerable proportion of unusable data, due to response patterns suggesting misinterpretation of the scale (i.e., interpreting the items of the BSCQ as reflecting likelihood of drinking rather than perceived ability to resist drinking urges in high-risk situations). These issues were addressed through rigorous data cleaning procedures and by excluding invalid or extreme cases from the analysis.

Second, while the sample size was large and the follow-up retention rate was generally high, the study was confined to adolescents enrolled in secondary schools in a specific region of Spain. This may limit the generalizability of the findings to other populations or educational contexts. Finally, while past-month cannabis use was assessed categorically (presence/absence), more nuanced patterns (e.g., frequency, context, or co-use severity) were not considered and could provide greater insight in future research.

Notwithstanding these limitations, this study contributes to the growing body of research examining individual differences in response to brief school-based interventions for alcohol use. Our findings, based on a profile-based approach, suggest that the GSC intervention may lead to a reduction in alcohol consumption in the short and medium term, as well as an improvement in self-efficacy in the short term, even among adolescents with varying histories of cannabis use. Furthermore, future studies should aim to identify the factors that help sustain these initial changes over time, especially in high-risk groups, to maximize the long-term impact of brief interventions in school settings.

## Data Availability

The datasets presented in this article are not readily available because they include information from underage participants collected under strict ethical conditions. The informed consent process specified that the data would be used exclusively for this specific research project and would not be shared with third parties. External access, including via repositories, is not permitted under the ethical approval granted, and would violate the legal and ethical commitments established with participants and their guardians. The study fully complies with the General Data Protection Regulation (GDPR) and national data protection laws in Spain. Request for further information regarding the study may be directed to José Luis Carballo (jcarballo@umh.es).
